# Supplementary material for: Pyruvate Dehydrogenase Kinase 1 inhibition mediated oxidative phosphorylation enhancement in cartilage promotes osteoarthritis progression
Source: BMC Musculoskelet Disord. 2023 Jul 20;24:597. doi: 10.1186/s12891-023-06585-6 (PMC10357736; doi:10.1186/s12891-023-06585-6)
Supplement: Supplementary file 1 — Additional file 1. [file 12891_2023_6585_MOESM1_ESM.zip › MF.Gsea.1653627316242/gsea_report_for_OA_1653627316242.html]

Report for OA 1653627316242 [GSEA]

| GS  follow link to MSigDB | GS DETAILS | SIZE | ES | NES | NOM p-val | FDR q-val | FWER p-val | RANK AT MAX | LEADING EDGE || 1 | GOMF\_OXIDOREDUCTION\_DRIVEN\_ACTIVE\_TRANSMEMBRANE\_TRANSPORTER\_ACTIVITY | Details ... | 52 | 0.68 | 2.78 | 0.000 | 0.000 | 0.000 | 2623 | tags=73%, list=20%, signal=91% |
| 2 | GOMF\_OXIDOREDUCTASE\_ACTIVITY\_ACTING\_ON\_NAD\_P\_H\_QUINONE\_OR\_SIMILAR\_COMPOUND\_AS\_ACCEPTOR | Details ... | 44 | 0.70 | 2.78 | 0.000 | 0.000 | 0.000 | 2708 | tags=80%, list=21%, signal=100% |
| 3 | GOMF\_NADH\_DEHYDROGENASE\_ACTIVITY | Details ... | 35 | 0.72 | 2.72 | 0.000 | 0.000 | 0.000 | 1838 | tags=77%, list=14%, signal=90% |
| 4 | GOMF\_OXIDOREDUCTASE\_ACTIVITY\_ACTING\_ON\_NAD\_P\_H | Details ... | 72 | 0.60 | 2.63 | 0.000 | 0.000 | 0.000 | 2708 | tags=64%, list=21%, signal=80% |
| 5 | GOMF\_ELECTRON\_TRANSFER\_ACTIVITY | Details ... | 104 | 0.55 | 2.51 | 0.000 | 0.000 | 0.000 | 3209 | tags=60%, list=25%, signal=79% |
| 6 | GOMF\_PROTON\_TRANSMEMBRANE\_TRANSPORTER\_ACTIVITY | Details ... | 88 | 0.52 | 2.38 | 0.000 | 0.000 | 0.000 | 3024 | tags=51%, list=23%, signal=66% |
| 7 | GOMF\_CATALYTIC\_ACTIVITY\_ACTING\_ON\_A\_GLYCOPROTEIN | Details ... | 17 | 0.69 | 2.13 | 0.000 | 0.002 | 0.018 | 2035 | tags=53%, list=16%, signal=63% |
| 8 | GOMF\_GLUTATHIONE\_TRANSFERASE\_ACTIVITY | Details ... | 22 | 0.65 | 2.12 | 0.000 | 0.002 | 0.020 | 1665 | tags=55%, list=13%, signal=63% |
| 9 | GOMF\_PRIMARY\_ACTIVE\_TRANSMEMBRANE\_TRANSPORTER\_ACTIVITY | Details ... | 140 | 0.42 | 2.04 | 0.000 | 0.006 | 0.062 | 3072 | tags=44%, list=24%, signal=57% |
| 10 | GOMF\_NUCLEOBASE\_CONTAINING\_COMPOUND\_KINASE\_ACTIVITY | Details ... | 30 | 0.55 | 2.02 | 0.000 | 0.007 | 0.077 | 1761 | tags=43%, list=14%, signal=50% |
| 11 | GOMF\_INTRAMOLECULAR\_OXIDOREDUCTASE\_ACTIVITY | Details ... | 43 | 0.51 | 2.01 | 0.000 | 0.008 | 0.092 | 2073 | tags=37%, list=16%, signal=44% |
| 12 | GOMF\_PYROPHOSPHATE\_HYDROLYSIS\_DRIVEN\_PROTON\_TRANSMEMBRANE\_TRANSPORTER\_ACTIVITY | Details ... | 16 | 0.65 | 1.98 | 0.002 | 0.010 | 0.128 | 3160 | tags=75%, list=24%, signal=99% |
| 13 | GOMF\_2\_IRON\_2\_SULFUR\_CLUSTER\_BINDING | Details ... | 16 | 0.64 | 1.91 | 0.002 | 0.020 | 0.246 | 1818 | tags=44%, list=14%, signal=51% |
| 14 | GOMF\_IMMUNOGLOBULIN\_RECEPTOR\_BINDING | Details ... | 26 | 0.54 | 1.89 | 0.000 | 0.022 | 0.283 | 3449 | tags=65%, list=27%, signal=89% |
| 15 | GOMF\_OXIDOREDUCTASE\_ACTIVITY\_ACTING\_ON\_A\_SULFUR\_GROUP\_OF\_DONORS | Details ... | 45 | 0.48 | 1.89 | 0.000 | 0.022 | 0.295 | 2434 | tags=33%, list=19%, signal=41% |
| 16 | GOMF\_PROTON\_CHANNEL\_ACTIVITY | Details ... | 15 | 0.64 | 1.86 | 0.002 | 0.026 | 0.362 | 3024 | tags=60%, list=23%, signal=78% |
| 17 | GOMF\_PROTEIN\_DISULFIDE\_ISOMERASE\_ACTIVITY | Details ... | 16 | 0.60 | 1.85 | 0.002 | 0.028 | 0.399 | 1175 | tags=38%, list=9%, signal=41% |
| 18 | GOMF\_GLUTATHIONE\_PEROXIDASE\_ACTIVITY | Details ... | 17 | 0.58 | 1.83 | 0.000 | 0.033 | 0.473 | 1919 | tags=53%, list=15%, signal=62% |
| 19 | GOMF\_THREONINE\_TYPE\_PEPTIDASE\_ACTIVITY | Details ... | 18 | 0.57 | 1.79 | 0.009 | 0.043 | 0.596 | 2597 | tags=44%, list=20%, signal=56% |
| 20 | GOMF\_NADPH\_BINDING | Details ... | 15 | 0.59 | 1.76 | 0.012 | 0.059 | 0.715 | 3640 | tags=67%, list=28%, signal=93% |
| 21 | GOMF\_ANTIGEN\_BINDING | Details ... | 84 | 0.38 | 1.73 | 0.000 | 0.070 | 0.793 | 3599 | tags=54%, list=28%, signal=74% |
| 22 | GOMF\_NAD\_BINDING | Details ... | 50 | 0.41 | 1.68 | 0.002 | 0.099 | 0.895 | 2031 | tags=32%, list=16%, signal=38% |
| 23 | GOMF\_CARBOXYPEPTIDASE\_ACTIVITY | Details ... | 34 | 0.45 | 1.65 | 0.015 | 0.121 | 0.937 | 2014 | tags=32%, list=16%, signal=38% |
| 24 | GOMF\_ALCOHOL\_DEHYDROGENASE\_NADPPLUS\_ACTIVITY | Details ... | 15 | 0.54 | 1.63 | 0.027 | 0.138 | 0.966 | 4102 | tags=53%, list=32%, signal=78% |
| 25 | GOMF\_STRUCTURAL\_CONSTITUENT\_OF\_MUSCLE | Details ... | 40 | 0.42 | 1.63 | 0.012 | 0.133 | 0.968 | 3529 | tags=55%, list=27%, signal=75% |
| 26 | GOMF\_PHOSPHOTRANSFERASE\_ACTIVITY\_PHOSPHATE\_GROUP\_AS\_ACCEPTOR | Details ... | 28 | 0.46 | 1.62 | 0.012 | 0.134 | 0.974 | 1761 | tags=32%, list=14%, signal=37% |
| 27 | GOMF\_PROTEIN\_CARRIER\_CHAPERONE | Details ... | 15 | 0.53 | 1.61 | 0.016 | 0.135 | 0.979 | 2839 | tags=60%, list=22%, signal=77% |
| 28 | GOMF\_LYASE\_ACTIVITY | Details ... | 163 | 0.32 | 1.60 | 0.000 | 0.145 | 0.984 | 3219 | tags=34%, list=25%, signal=45% |
| 29 | GOMF\_O\_METHYLTRANSFERASE\_ACTIVITY | Details ... | 17 | 0.51 | 1.60 | 0.034 | 0.140 | 0.984 | 2664 | tags=53%, list=21%, signal=67% |
| 30 | GOMF\_OXIDOREDUCTASE\_ACTIVITY\_ACTING\_ON\_THE\_ALDEHYDE\_OR\_OXO\_GROUP\_OF\_DONORS\_NAD\_OR\_NADP\_AS\_ACCEPTOR | Details ... | 34 | 0.43 | 1.59 | 0.020 | 0.144 | 0.988 | 3138 | tags=44%, list=24%, signal=58% |
| 31 | GOMF\_DISULFIDE\_OXIDOREDUCTASE\_ACTIVITY | Details ... | 34 | 0.43 | 1.59 | 0.015 | 0.144 | 0.989 | 2073 | tags=26%, list=16%, signal=31% |
| 32 | GOMF\_VITAMIN\_BINDING | Details ... | 120 | 0.33 | 1.58 | 0.000 | 0.146 | 0.990 | 3632 | tags=42%, list=28%, signal=57% |
| 33 | GOMF\_NUCLEOTIDE\_DIPHOSPHATASE\_ACTIVITY | Details ... | 15 | 0.52 | 1.57 | 0.043 | 0.148 | 0.991 | 3751 | tags=67%, list=29%, signal=94% |
| 34 | GOMF\_GALACTOSYLTRANSFERASE\_ACTIVITY | Details ... | 25 | 0.46 | 1.57 | 0.028 | 0.145 | 0.993 | 1500 | tags=32%, list=12%, signal=36% |
| 35 | GOMF\_CARBON\_CARBON\_LYASE\_ACTIVITY | Details ... | 40 | 0.41 | 1.57 | 0.014 | 0.143 | 0.993 | 1870 | tags=35%, list=14%, signal=41% |
| 36 | GOMF\_OXIDOREDUCTASE\_ACTIVITY\_ACTING\_ON\_THE\_ALDEHYDE\_OR\_OXO\_GROUP\_OF\_DONORS | Details ... | 42 | 0.39 | 1.53 | 0.026 | 0.182 | 0.999 | 3493 | tags=45%, list=27%, signal=62% |
| 37 | GOMF\_DAMAGED\_DNA\_BINDING | Details ... | 64 | 0.36 | 1.52 | 0.010 | 0.187 | 1.000 | 1696 | tags=28%, list=13%, signal=32% |
| 38 | GOMF\_ATPASE\_COUPLED\_CATION\_TRANSMEMBRANE\_TRANSPORTER\_ACTIVITY | Details ... | 38 | 0.40 | 1.51 | 0.040 | 0.190 | 1.000 | 3263 | tags=45%, list=25%, signal=60% |
| 39 | GOMF\_WNT\_PROTEIN\_BINDING | Details ... | 24 | 0.46 | 1.51 | 0.031 | 0.190 | 1.000 | 2503 | tags=42%, list=19%, signal=52% |
| 40 | GOMF\_THIOLESTER\_HYDROLASE\_ACTIVITY | Details ... | 27 | 0.43 | 1.49 | 0.037 | 0.216 | 1.000 | 1526 | tags=33%, list=12%, signal=38% |
| 41 | GOMF\_ALDO\_KETO\_REDUCTASE\_NADP\_ACTIVITY | Details ... | 20 | 0.46 | 1.48 | 0.062 | 0.215 | 1.000 | 4102 | tags=50%, list=32%, signal=73% |
| 42 | GOMF\_CARBOXY\_LYASE\_ACTIVITY | Details ... | 27 | 0.41 | 1.44 | 0.054 | 0.275 | 1.000 | 2936 | tags=44%, list=23%, signal=57% |
| 43 | GOMF\_C\_ACYLTRANSFERASE\_ACTIVITY | Details ... | 16 | 0.47 | 1.43 | 0.091 | 0.280 | 1.000 | 2913 | tags=44%, list=23%, signal=56% |
| 44 | GOMF\_COA\_HYDROLASE\_ACTIVITY | Details ... | 16 | 0.47 | 1.41 | 0.084 | 0.313 | 1.000 | 3031 | tags=50%, list=23%, signal=65% |
| 45 | GOMF\_HEXOSYLTRANSFERASE\_ACTIVITY | Details ... | 133 | 0.29 | 1.40 | 0.026 | 0.321 | 1.000 | 3641 | tags=42%, list=28%, signal=58% |
| 46 | GOMF\_ACETYLGALACTOSAMINYLTRANSFERASE\_ACTIVITY | Details ... | 20 | 0.42 | 1.39 | 0.072 | 0.334 | 1.000 | 2954 | tags=40%, list=23%, signal=52% |
| 47 | GOMF\_TRANSFERASE\_ACTIVITY\_TRANSFERRING\_ALKYL\_OR\_ARYL\_OTHER\_THAN\_METHYL\_GROUPS | Details ... | 52 | 0.34 | 1.39 | 0.035 | 0.342 | 1.000 | 1884 | tags=31%, list=15%, signal=36% |
| 48 | GOMF\_FLAVIN\_ADENINE\_DINUCLEOTIDE\_BINDING | Details ... | 76 | 0.31 | 1.38 | 0.037 | 0.344 | 1.000 | 2231 | tags=30%, list=17%, signal=36% |
| 49 | GOMF\_SINGLE\_STRANDED\_DNA\_HELICASE\_ACTIVITY | Details ... | 17 | 0.45 | 1.38 | 0.096 | 0.345 | 1.000 | 1581 | tags=41%, list=12%, signal=47% |
| 50 | GOMF\_DNA\_REPLICATION\_ORIGIN\_BINDING | Details ... | 16 | 0.44 | 1.37 | 0.088 | 0.351 | 1.000 | 1410 | tags=50%, list=11%, signal=56% |
| 51 | GOMF\_ACETYLGLUCOSAMINYLTRANSFERASE\_ACTIVITY | Details ... | 35 | 0.36 | 1.36 | 0.083 | 0.370 | 1.000 | 2646 | tags=34%, list=21%, signal=43% |
| 52 | GOMF\_OXIDOREDUCTASE\_ACTIVITY\_ACTING\_ON\_THE\_CH\_CH\_GROUP\_OF\_DONORS | Details ... | 49 | 0.33 | 1.35 | 0.069 | 0.376 | 1.000 | 3382 | tags=41%, list=26%, signal=55% |
| 53 | GOMF\_EXOPEPTIDASE\_ACTIVITY | Details ... | 83 | 0.30 | 1.35 | 0.043 | 0.369 | 1.000 | 2014 | tags=24%, list=16%, signal=28% |
| 54 | GOMF\_GLUCURONOSYLTRANSFERASE\_ACTIVITY | Details ... | 19 | 0.42 | 1.35 | 0.106 | 0.362 | 1.000 | 3321 | tags=53%, list=26%, signal=71% |
| 55 | GOMF\_OXIDOREDUCTASE\_ACTIVITY\_ACTING\_ON\_PEROXIDE\_AS\_ACCEPTOR | Details ... | 42 | 0.35 | 1.35 | 0.063 | 0.359 | 1.000 | 2161 | tags=38%, list=17%, signal=46% |
| 56 | GOMF\_L\_ASCORBIC\_ACID\_BINDING | Details ... | 16 | 0.45 | 1.35 | 0.122 | 0.353 | 1.000 | 3632 | tags=56%, list=28%, signal=78% |
| 57 | GOMF\_HYDROLASE\_ACTIVITY\_ACTING\_ON\_GLYCOSYL\_BONDS | Details ... | 109 | 0.29 | 1.34 | 0.040 | 0.359 | 1.000 | 1767 | tags=26%, list=14%, signal=30% |
| 58 | GOMF\_METAL\_CLUSTER\_BINDING | Details ... | 56 | 0.32 | 1.33 | 0.076 | 0.383 | 1.000 | 4161 | tags=57%, list=32%, signal=84% |
| 59 | GOMF\_UDP\_GLYCOSYLTRANSFERASE\_ACTIVITY | Details ... | 97 | 0.29 | 1.33 | 0.040 | 0.385 | 1.000 | 3442 | tags=38%, list=27%, signal=52% |
| 60 | GOMF\_FATTY\_ACID\_DERIVATIVE\_BINDING | Details ... | 16 | 0.44 | 1.32 | 0.133 | 0.387 | 1.000 | 971 | tags=19%, list=8%, signal=20% |
| 61 | GOMF\_GLYCOLIPID\_BINDING | Details ... | 24 | 0.39 | 1.32 | 0.106 | 0.389 | 1.000 | 1675 | tags=25%, list=13%, signal=29% |
| 62 | GOMF\_S\_ADENOSYL\_L\_METHIONINE\_BINDING | Details ... | 15 | 0.45 | 1.31 | 0.160 | 0.403 | 1.000 | 330 | tags=13%, list=3%, signal=14% |
| 63 | GOMF\_TRANSFORMING\_GROWTH\_FACTOR\_BETA\_RECEPTOR\_BINDING | Details ... | 22 | 0.40 | 1.31 | 0.136 | 0.399 | 1.000 | 2490 | tags=27%, list=19%, signal=34% |
| 64 | GOMF\_ANTIOXIDANT\_ACTIVITY | Details ... | 64 | 0.30 | 1.30 | 0.099 | 0.409 | 1.000 | 2776 | tags=38%, list=22%, signal=48% |
| 65 | GOMF\_COPPER\_ION\_BINDING | Details ... | 47 | 0.33 | 1.30 | 0.115 | 0.407 | 1.000 | 2413 | tags=36%, list=19%, signal=44% |
| 66 | GOMF\_OXIDOREDUCTASE\_ACTIVITY\_ACTING\_ON\_THE\_CH\_NH2\_GROUP\_OF\_DONORS | Details ... | 16 | 0.44 | 1.30 | 0.142 | 0.405 | 1.000 | 2192 | tags=44%, list=17%, signal=53% |
| 67 | GOMF\_CATALYTIC\_ACTIVITY\_ACTING\_ON\_A\_TRNA | Details ... | 88 | 0.28 | 1.29 | 0.058 | 0.410 | 1.000 | 1913 | tags=25%, list=15%, signal=29% |
| 68 | GOMF\_VITAMIN\_B6\_BINDING | Details ... | 45 | 0.33 | 1.29 | 0.112 | 0.405 | 1.000 | 2534 | tags=31%, list=20%, signal=39% |
| 69 | GOMF\_HYDROLASE\_ACTIVITY\_HYDROLYZING\_O\_GLYCOSYL\_COMPOUNDS | Details ... | 66 | 0.30 | 1.28 | 0.086 | 0.414 | 1.000 | 2360 | tags=32%, list=18%, signal=39% |
| 70 | GOMF\_UBIQUITIN\_LIGASE\_SUBSTRATE\_ADAPTOR\_ACTIVITY | Details ... | 19 | 0.40 | 1.28 | 0.165 | 0.409 | 1.000 | 126 | tags=16%, list=1%, signal=16% |
| 71 | GOMF\_NADPLUS\_BINDING | Details ... | 16 | 0.42 | 1.27 | 0.163 | 0.427 | 1.000 | 4477 | tags=56%, list=35%, signal=86% |
| 72 | GOMF\_ISOPEPTIDASE\_ACTIVITY | Details ... | 16 | 0.41 | 1.26 | 0.191 | 0.461 | 1.000 | 3117 | tags=44%, list=24%, signal=58% |
| 73 | GOMF\_CARBON\_OXYGEN\_LYASE\_ACTIVITY | Details ... | 69 | 0.29 | 1.25 | 0.110 | 0.469 | 1.000 | 1925 | tags=20%, list=15%, signal=24% |
| 74 | GOMF\_POLYSACCHARIDE\_BINDING | Details ... | 15 | 0.41 | 1.24 | 0.168 | 0.479 | 1.000 | 708 | tags=20%, list=5%, signal=21% |
| 75 | GOMF\_HYDROLASE\_ACTIVITY\_ACTING\_ON\_CARBON\_NITROGEN\_BUT\_NOT\_PEPTIDE\_BONDS | Details ... | 100 | 0.27 | 1.24 | 0.104 | 0.477 | 1.000 | 1955 | tags=27%, list=15%, signal=32% |
| 76 | GOMF\_IMMUNOGLOBULIN\_BINDING | Details ... | 23 | 0.38 | 1.24 | 0.186 | 0.478 | 1.000 | 2752 | tags=43%, list=21%, signal=55% |
| 77 | GOMF\_OXIDOREDUCTASE\_ACTIVITY\_ACTING\_ON\_THE\_CH\_CH\_GROUP\_OF\_DONORS\_NAD\_OR\_NADP\_AS\_ACCEPTOR | Details ... | 23 | 0.36 | 1.24 | 0.182 | 0.478 | 1.000 | 4410 | tags=61%, list=34%, signal=92% |
| 78 | GOMF\_HYDRO\_LYASE\_ACTIVITY | Details ... | 54 | 0.30 | 1.24 | 0.152 | 0.474 | 1.000 | 2215 | tags=24%, list=17%, signal=29% |
| 79 | GOMF\_WNT\_ACTIVATED\_RECEPTOR\_ACTIVITY | Details ... | 16 | 0.40 | 1.22 | 0.196 | 0.504 | 1.000 | 1990 | tags=25%, list=15%, signal=30% |
| 80 | GOMF\_SINGLE\_STRANDED\_DNA\_BINDING | Details ... | 106 | 0.26 | 1.22 | 0.132 | 0.502 | 1.000 | 1805 | tags=23%, list=14%, signal=26% |
| 81 | GOMF\_ACTIVE\_TRANSMEMBRANE\_TRANSPORTER\_ACTIVITY | Details ... | 303 | 0.22 | 1.21 | 0.042 | 0.521 | 1.000 | 3072 | tags=31%, list=24%, signal=40% |
| 82 | GOMF\_PEPTIDASE\_ACTIVATOR\_ACTIVITY\_INVOLVED\_IN\_APOPTOTIC\_PROCESS | Details ... | 19 | 0.38 | 1.21 | 0.212 | 0.515 | 1.000 | 1164 | tags=32%, list=9%, signal=35% |
| 83 | GOMF\_PROTEIN\_PHOSPHATASE\_1\_BINDING | Details ... | 19 | 0.38 | 1.20 | 0.230 | 0.545 | 1.000 | 1002 | tags=32%, list=8%, signal=34% |
| 84 | GOMF\_ISOMERASE\_ACTIVITY | Details ... | 124 | 0.25 | 1.18 | 0.101 | 0.580 | 1.000 | 2398 | tags=27%, list=19%, signal=32% |
| 85 | GOMF\_CULLIN\_FAMILY\_PROTEIN\_BINDING | Details ... | 18 | 0.38 | 1.17 | 0.226 | 0.608 | 1.000 | 2302 | tags=33%, list=18%, signal=41% |
| 86 | GOMF\_TRANSFERASE\_ACTIVITY\_TRANSFERRING\_NITROGENOUS\_GROUPS | Details ... | 17 | 0.37 | 1.17 | 0.261 | 0.616 | 1.000 | 2074 | tags=24%, list=16%, signal=28% |
| 87 | GOMF\_LIGASE\_ACTIVITY | Details ... | 129 | 0.24 | 1.17 | 0.145 | 0.610 | 1.000 | 3024 | tags=32%, list=23%, signal=41% |
| 88 | GOMF\_GLYCOSYLTRANSFERASE\_ACTIVITY | Details ... | 188 | 0.23 | 1.17 | 0.123 | 0.605 | 1.000 | 3667 | tags=40%, list=28%, signal=55% |
| 89 | GOMF\_OXIDOREDUCTASE\_ACTIVITY\_ACTING\_ON\_CH\_OH\_GROUP\_OF\_DONORS | Details ... | 107 | 0.24 | 1.16 | 0.149 | 0.602 | 1.000 | 967 | tags=15%, list=7%, signal=16% |
| 90 | GOMF\_MODIFIED\_AMINO\_ACID\_BINDING | Details ... | 73 | 0.26 | 1.16 | 0.187 | 0.603 | 1.000 | 3144 | tags=33%, list=24%, signal=43% |
| 91 | GOMF\_CARBOHYDRATE\_KINASE\_ACTIVITY | Details ... | 21 | 0.35 | 1.16 | 0.210 | 0.601 | 1.000 | 256 | tags=14%, list=2%, signal=15% |
| 92 | GOMF\_RECEPTOR\_ANTAGONIST\_ACTIVITY | Details ... | 17 | 0.37 | 1.16 | 0.261 | 0.601 | 1.000 | 809 | tags=18%, list=6%, signal=19% |
| 93 | GOMF\_TRANSFERASE\_ACTIVITY\_TRANSFERRING\_ONE\_CARBON\_GROUPS | Details ... | 158 | 0.23 | 1.15 | 0.166 | 0.613 | 1.000 | 1885 | tags=20%, list=15%, signal=23% |
| 94 | GOMF\_RNA\_POLYMERASE\_ACTIVITY | Details ... | 33 | 0.30 | 1.15 | 0.235 | 0.615 | 1.000 | 1760 | tags=30%, list=14%, signal=35% |
| 95 | GOMF\_HISTONE\_ACETYLTRANSFERASE\_BINDING | Details ... | 21 | 0.35 | 1.15 | 0.273 | 0.616 | 1.000 | 1557 | tags=29%, list=12%, signal=32% |
| 96 | GOMF\_FRIZZLED\_BINDING | Details ... | 30 | 0.32 | 1.14 | 0.252 | 0.616 | 1.000 | 2700 | tags=37%, list=21%, signal=46% |
| 97 | GOMF\_OXIDOREDUCTASE\_ACTIVITY\_ACTING\_ON\_SINGLE\_DONORS\_WITH\_INCORPORATION\_OF\_MOLECULAR\_OXYGEN | Details ... | 21 | 0.34 | 1.13 | 0.290 | 0.646 | 1.000 | 3209 | tags=48%, list=25%, signal=63% |
| 98 | GOMF\_LOW\_DENSITY\_LIPOPROTEIN\_PARTICLE\_RECEPTOR\_BINDING | Details ... | 20 | 0.35 | 1.13 | 0.290 | 0.641 | 1.000 | 2083 | tags=35%, list=16%, signal=42% |
| 99 | GOMF\_CARBOXYLIC\_ESTER\_HYDROLASE\_ACTIVITY | Details ... | 99 | 0.24 | 1.13 | 0.194 | 0.640 | 1.000 | 1836 | tags=19%, list=14%, signal=22% |
| 100 | GOMF\_NUCLEASE\_ACTIVITY | Details ... | 152 | 0.22 | 1.12 | 0.184 | 0.655 | 1.000 | 3488 | tags=38%, list=27%, signal=52% |
| 101 | GOMF\_PHOSPHOLIPASE\_C\_ACTIVITY |  | 23 | 0.33 | 1.12 | 0.253 | 0.657 | 1.000 | 2617 | tags=30%, list=20%, signal=38% |
| 102 | GOMF\_PROTEIN\_MEMBRANE\_ADAPTOR\_ACTIVITY |  | 23 | 0.33 | 1.12 | 0.293 | 0.655 | 1.000 | 436 | tags=13%, list=3%, signal=13% |
| 103 | GOMF\_SOLUTE\_PROTON\_ANTIPORTER\_ACTIVITY |  | 15 | 0.38 | 1.11 | 0.314 | 0.664 | 1.000 | 2817 | tags=47%, list=22%, signal=60% |
| 104 | GOMF\_SNAP\_RECEPTOR\_ACTIVITY |  | 31 | 0.31 | 1.11 | 0.296 | 0.657 | 1.000 | 2901 | tags=32%, list=22%, signal=42% |
| 105 | GOMF\_G\_PROTEIN\_ALPHA\_SUBUNIT\_BINDING |  | 21 | 0.33 | 1.10 | 0.318 | 0.684 | 1.000 | 2842 | tags=38%, list=22%, signal=49% |
| 106 | GOMF\_PEPTIDASE\_ACTIVATOR\_ACTIVITY |  | 39 | 0.29 | 1.09 | 0.316 | 0.732 | 1.000 | 3882 | tags=54%, list=30%, signal=77% |
| 107 | GOMF\_MANNOSE\_BINDING |  | 18 | 0.35 | 1.08 | 0.352 | 0.739 | 1.000 | 1856 | tags=28%, list=14%, signal=32% |
| 108 | GOMF\_S\_ADENOSYLMETHIONINE\_DEPENDENT\_METHYLTRANSFERASE\_ACTIVITY |  | 112 | 0.22 | 1.07 | 0.296 | 0.762 | 1.000 | 1885 | tags=20%, list=15%, signal=23% |
| 109 | GOMF\_ENDORIBONUCLEASE\_ACTIVITY\_PRODUCING\_5\_PHOSPHOMONOESTERS |  | 25 | 0.31 | 1.07 | 0.349 | 0.775 | 1.000 | 2091 | tags=36%, list=16%, signal=43% |
| 110 | GOMF\_4\_IRON\_4\_SULFUR\_CLUSTER\_BINDING |  | 38 | 0.28 | 1.06 | 0.362 | 0.791 | 1.000 | 3830 | tags=55%, list=30%, signal=78% |
| 111 | GOMF\_LIPASE\_ACTIVITY |  | 92 | 0.23 | 1.05 | 0.332 | 0.805 | 1.000 | 2740 | tags=26%, list=21%, signal=33% |
| 112 | GOMF\_PHOSPHOLIPASE\_BINDING |  | 22 | 0.32 | 1.05 | 0.390 | 0.813 | 1.000 | 2760 | tags=45%, list=21%, signal=58% |
| 113 | GOMF\_LIPASE\_INHIBITOR\_ACTIVITY |  | 18 | 0.34 | 1.05 | 0.383 | 0.812 | 1.000 | 1855 | tags=28%, list=14%, signal=32% |
| 114 | GOMF\_TRANSLATION\_FACTOR\_ACTIVITY\_RNA\_BINDING |  | 68 | 0.24 | 1.05 | 0.372 | 0.805 | 1.000 | 1301 | tags=19%, list=10%, signal=21% |
| 115 | GOMF\_METALLOEXOPEPTIDASE\_ACTIVITY |  | 57 | 0.25 | 1.05 | 0.371 | 0.805 | 1.000 | 1918 | tags=21%, list=15%, signal=25% |
| 116 | GOMF\_DEAMINASE\_ACTIVITY |  | 23 | 0.31 | 1.04 | 0.386 | 0.814 | 1.000 | 1955 | tags=35%, list=15%, signal=41% |
| 117 | GOMF\_METALLOCARBOXYPEPTIDASE\_ACTIVITY |  | 21 | 0.32 | 1.04 | 0.386 | 0.808 | 1.000 | 1918 | tags=24%, list=15%, signal=28% |
| 118 | GOMF\_SODIUM\_CHANNEL\_REGULATOR\_ACTIVITY |  | 29 | 0.30 | 1.04 | 0.390 | 0.811 | 1.000 | 660 | tags=14%, list=5%, signal=15% |
| 119 | GOMF\_SULFUR\_COMPOUND\_BINDING |  | 221 | 0.20 | 1.04 | 0.334 | 0.808 | 1.000 | 2629 | tags=24%, list=20%, signal=30% |
| 120 | GOMF\_G\_PROTEIN\_COUPLED\_CHEMOATTRACTANT\_RECEPTOR\_ACTIVITY |  | 26 | 0.31 | 1.04 | 0.384 | 0.805 | 1.000 | 3421 | tags=35%, list=27%, signal=47% |
| 121 | GOMF\_NADP\_BINDING |  | 49 | 0.26 | 1.03 | 0.379 | 0.808 | 1.000 | 4613 | tags=63%, list=36%, signal=98% |
| 122 | GOMF\_CYCLASE\_ACTIVITY |  | 20 | 0.32 | 1.03 | 0.428 | 0.804 | 1.000 | 2982 | tags=35%, list=23%, signal=45% |
| 123 | GOMF\_MONOSACCHARIDE\_BINDING |  | 61 | 0.24 | 1.03 | 0.388 | 0.800 | 1.000 | 1532 | tags=16%, list=12%, signal=19% |
| 124 | GOMF\_ATPASE\_BINDING |  | 72 | 0.23 | 1.03 | 0.402 | 0.812 | 1.000 | 3855 | tags=40%, list=30%, signal=57% |
| 125 | GOMF\_ANION\_CHANNEL\_ACTIVITY |  | 66 | 0.24 | 1.03 | 0.385 | 0.806 | 1.000 | 2087 | tags=24%, list=16%, signal=29% |
| 126 | GOMF\_GDP\_BINDING |  | 60 | 0.24 | 1.03 | 0.385 | 0.804 | 1.000 | 2339 | tags=23%, list=18%, signal=28% |
| 127 | GOMF\_CALCIUM\_DEPENDENT\_PROTEIN\_BINDING |  | 74 | 0.23 | 1.02 | 0.422 | 0.806 | 1.000 | 3097 | tags=31%, list=24%, signal=41% |
| 128 | GOMF\_LIPOPOLYSACCHARIDE\_BINDING |  | 26 | 0.30 | 1.02 | 0.445 | 0.805 | 1.000 | 4573 | tags=58%, list=35%, signal=89% |
| 129 | GOMF\_ADP\_BINDING |  | 33 | 0.27 | 1.02 | 0.406 | 0.800 | 1.000 | 2778 | tags=36%, list=22%, signal=46% |
| 130 | GOMF\_GLUCOSYLTRANSFERASE\_ACTIVITY |  | 16 | 0.33 | 1.02 | 0.427 | 0.797 | 1.000 | 1209 | tags=25%, list=9%, signal=28% |
| 131 | GOMF\_FATTY\_ACID\_BINDING |  | 41 | 0.26 | 1.02 | 0.401 | 0.797 | 1.000 | 3661 | tags=39%, list=28%, signal=54% |
| 132 | GOMF\_METALLOAMINOPEPTIDASE\_ACTIVITY |  | 23 | 0.31 | 1.02 | 0.406 | 0.792 | 1.000 | 634 | tags=17%, list=5%, signal=18% |
| 133 | GOMF\_PHOSPHATIDYLINOSITOL\_BINDING |  | 199 | 0.19 | 1.01 | 0.418 | 0.798 | 1.000 | 1706 | tags=16%, list=13%, signal=18% |
| 134 | GOMF\_DNA\_DIRECTED\_DNA\_POLYMERASE\_ACTIVITY |  | 18 | 0.32 | 1.01 | 0.421 | 0.818 | 1.000 | 820 | tags=17%, list=6%, signal=18% |
| 135 | GOMF\_HYDROLASE\_ACTIVITY\_HYDROLYZING\_N\_GLYCOSYL\_COMPOUNDS |  | 42 | 0.26 | 1.01 | 0.467 | 0.814 | 1.000 | 1069 | tags=19%, list=8%, signal=21% |
| 136 | GOMF\_HYDROLASE\_ACTIVITY\_ACTING\_ON\_CARBON\_NITROGEN\_BUT\_NOT\_PEPTIDE\_BONDS\_IN\_LINEAR\_AMIDES |  | 52 | 0.24 | 1.00 | 0.429 | 0.816 | 1.000 | 1758 | tags=25%, list=14%, signal=29% |
| 137 | GOMF\_PROTEIN\_SELF\_ASSOCIATION |  | 51 | 0.25 | 1.00 | 0.460 | 0.814 | 1.000 | 2862 | tags=29%, list=22%, signal=38% |
| 138 | GOMF\_CHANNEL\_REGULATOR\_ACTIVITY |  | 108 | 0.21 | 1.00 | 0.434 | 0.811 | 1.000 | 793 | tags=11%, list=6%, signal=12% |
| 139 | GOMF\_UBIQUITIN\_LIKE\_PROTEIN\_TRANSFERASE\_ACTIVITY |  | 288 | 0.18 | 1.00 | 0.460 | 0.807 | 1.000 | 3283 | tags=31%, list=25%, signal=41% |
| 140 | GOMF\_EXODEOXYRIBONUCLEASE\_ACTIVITY |  | 20 | 0.31 | 1.00 | 0.440 | 0.802 | 1.000 | 2166 | tags=35%, list=17%, signal=42% |
| 141 | GOMF\_FAD\_BINDING |  | 34 | 0.27 | 1.00 | 0.449 | 0.799 | 1.000 | 2446 | tags=29%, list=19%, signal=36% |
| 142 | GOMF\_TBP\_CLASS\_PROTEIN\_BINDING |  | 24 | 0.29 | 0.99 | 0.487 | 0.835 | 1.000 | 1987 | tags=29%, list=15%, signal=34% |
| 143 | GOMF\_TRANSLATION\_ELONGATION\_FACTOR\_ACTIVITY |  | 15 | 0.33 | 0.98 | 0.475 | 0.843 | 1.000 | 1120 | tags=27%, list=9%, signal=29% |
| 144 | GOMF\_PHOSPHOLIPASE\_ACTIVITY |  | 77 | 0.22 | 0.98 | 0.509 | 0.843 | 1.000 | 2656 | tags=25%, list=21%, signal=31% |
| 145 | GOMF\_PHOSPHORUS\_OXYGEN\_LYASE\_ACTIVITY |  | 21 | 0.30 | 0.98 | 0.466 | 0.839 | 1.000 | 2982 | tags=33%, list=23%, signal=43% |
| 146 | GOMF\_UBIQUITIN\_LIKE\_PROTEIN\_CONJUGATING\_ENZYME\_ACTIVITY |  | 28 | 0.28 | 0.98 | 0.467 | 0.839 | 1.000 | 1651 | tags=25%, list=13%, signal=29% |
| 147 | GOMF\_G\_PROTEIN\_BETA\_SUBUNIT\_BINDING |  | 15 | 0.33 | 0.97 | 0.481 | 0.847 | 1.000 | 8631 | tags=100%, list=67%, signal=302% |
| 148 | GOMF\_PROTEIN\_METHYLTRANSFERASE\_ACTIVITY |  | 65 | 0.23 | 0.97 | 0.519 | 0.843 | 1.000 | 1359 | tags=18%, list=11%, signal=21% |
| 149 | GOMF\_LIPOPROTEIN\_PARTICLE\_RECEPTOR\_BINDING |  | 25 | 0.28 | 0.97 | 0.487 | 0.847 | 1.000 | 2083 | tags=28%, list=16%, signal=33% |
| 150 | GOMF\_NUCLEOTIDYLTRANSFERASE\_ACTIVITY |  | 98 | 0.21 | 0.97 | 0.529 | 0.850 | 1.000 | 1760 | tags=18%, list=14%, signal=21% |
| 151 | GOMF\_ENDONUCLEASE\_ACTIVITY |  | 93 | 0.21 | 0.96 | 0.528 | 0.857 | 1.000 | 3488 | tags=39%, list=27%, signal=53% |
| 152 | GOMF\_PROTEIN\_TYROSINE\_KINASE\_ACTIVATOR\_ACTIVITY |  | 15 | 0.33 | 0.96 | 0.506 | 0.852 | 1.000 | 2516 | tags=27%, list=20%, signal=33% |
| 153 | GOMF\_AMINOPEPTIDASE\_ACTIVITY |  | 32 | 0.26 | 0.96 | 0.496 | 0.856 | 1.000 | 634 | tags=13%, list=5%, signal=13% |
| 154 | GOMF\_CIS\_TRANS\_ISOMERASE\_ACTIVITY |  | 35 | 0.26 | 0.96 | 0.529 | 0.856 | 1.000 | 2304 | tags=34%, list=18%, signal=42% |
| 155 | GOMF\_LIGAND\_GATED\_ANION\_CHANNEL\_ACTIVITY |  | 17 | 0.30 | 0.96 | 0.510 | 0.852 | 1.000 | 3350 | tags=41%, list=26%, signal=56% |
| 156 | GOMF\_MISFOLDED\_PROTEIN\_BINDING |  | 25 | 0.27 | 0.95 | 0.529 | 0.868 | 1.000 | 1139 | tags=20%, list=9%, signal=22% |
| 157 | GOMF\_CARGO\_ADAPTOR\_ACTIVITY |  | 15 | 0.33 | 0.95 | 0.526 | 0.864 | 1.000 | 1821 | tags=40%, list=14%, signal=47% |
| 158 | GOMF\_EXTRACELLULAR\_MATRIX\_STRUCTURAL\_CONSTITUENT\_CONFERRING\_COMPRESSION\_RESISTANCE |  | 19 | 0.30 | 0.95 | 0.523 | 0.858 | 1.000 | 3681 | tags=47%, list=29%, signal=66% |
| 159 | GOMF\_MANGANESE\_ION\_BINDING |  | 55 | 0.23 | 0.95 | 0.521 | 0.855 | 1.000 | 2249 | tags=27%, list=17%, signal=33% |
| 160 | GOMF\_CYSTEINE\_TYPE\_PEPTIDASE\_ACTIVITY |  | 133 | 0.19 | 0.95 | 0.585 | 0.852 | 1.000 | 1929 | tags=19%, list=15%, signal=22% |
| 161 | GOMF\_POTASSIUM\_CHANNEL\_REGULATOR\_ACTIVITY |  | 38 | 0.25 | 0.95 | 0.535 | 0.848 | 1.000 | 1361 | tags=18%, list=11%, signal=21% |
| 162 | GOMF\_PALMITOYLTRANSFERASE\_ACTIVITY |  | 21 | 0.29 | 0.95 | 0.529 | 0.855 | 1.000 | 1694 | tags=29%, list=13%, signal=33% |
| 163 | GOMF\_LIGASE\_ACTIVITY\_FORMING\_CARBON\_OXYGEN\_BONDS |  | 33 | 0.26 | 0.94 | 0.546 | 0.861 | 1.000 | 1913 | tags=24%, list=15%, signal=28% |
| 164 | GOMF\_ENDONUCLEASE\_ACTIVITY\_ACTIVE\_WITH\_EITHER\_RIBO\_OR\_DEOXYRIBONUCLEIC\_ACIDS\_AND\_PRODUCING\_5\_PHOSPHOMONOESTERS |  | 32 | 0.26 | 0.94 | 0.547 | 0.870 | 1.000 | 2091 | tags=31%, list=16%, signal=37% |
| 165 | GOMF\_PEPTIDASE\_REGULATOR\_ACTIVITY |  | 171 | 0.18 | 0.93 | 0.667 | 0.882 | 1.000 | 2161 | tags=19%, list=17%, signal=23% |
| 166 | GOMF\_ACYL\_COA\_BINDING |  | 19 | 0.29 | 0.93 | 0.567 | 0.886 | 1.000 | 971 | tags=16%, list=8%, signal=17% |
| 167 | GOMF\_DNA\_BINDING\_BENDING |  | 16 | 0.30 | 0.93 | 0.536 | 0.893 | 1.000 | 393 | tags=13%, list=3%, signal=13% |
| 168 | GOMF\_CARBOHYDRATE\_BINDING |  | 196 | 0.18 | 0.92 | 0.728 | 0.913 | 1.000 | 1586 | tags=14%, list=12%, signal=15% |
| 169 | GOMF\_HYDROLASE\_ACTIVITY\_ACTING\_ON\_CARBON\_NITROGEN\_BUT\_NOT\_PEPTIDE\_BONDS\_IN\_CYCLIC\_AMIDINES |  | 26 | 0.27 | 0.92 | 0.586 | 0.912 | 1.000 | 1955 | tags=31%, list=15%, signal=36% |
| 170 | GOMF\_LOW\_DENSITY\_LIPOPROTEIN\_PARTICLE\_BINDING |  | 15 | 0.30 | 0.91 | 0.574 | 0.915 | 1.000 | 457 | tags=13%, list=4%, signal=14% |
| 171 | GOMF\_RIBONUCLEASE\_ACTIVITY |  | 86 | 0.20 | 0.91 | 0.638 | 0.926 | 1.000 | 3488 | tags=34%, list=27%, signal=46% |
| 172 | GOMF\_TRIGLYCERIDE\_LIPASE\_ACTIVITY |  | 17 | 0.29 | 0.90 | 0.586 | 0.931 | 1.000 | 457 | tags=12%, list=4%, signal=12% |
| 173 | GOMF\_ATPASE\_COUPLED\_TRANSMEMBRANE\_TRANSPORTER\_ACTIVITY |  | 88 | 0.20 | 0.90 | 0.683 | 0.931 | 1.000 | 1304 | tags=13%, list=10%, signal=14% |
| 174 | GOMF\_ENDORIBONUCLEASE\_ACTIVITY |  | 51 | 0.22 | 0.90 | 0.642 | 0.928 | 1.000 | 3617 | tags=39%, list=28%, signal=54% |
| 175 | GOMF\_HEPARIN\_BINDING |  | 138 | 0.18 | 0.90 | 0.720 | 0.926 | 1.000 | 2519 | tags=25%, list=20%, signal=30% |
| 176 | GOMF\_TRANSMEMBRANE\_TRANSPORTER\_BINDING |  | 103 | 0.19 | 0.90 | 0.698 | 0.923 | 1.000 | 1679 | tags=17%, list=13%, signal=19% |
| 177 | GOMF\_G\_PROTEIN\_ACTIVITY |  | 34 | 0.24 | 0.89 | 0.618 | 0.929 | 1.000 | 2453 | tags=26%, list=19%, signal=33% |
| 178 | GOMF\_TRANSLATION\_INITIATION\_FACTOR\_BINDING |  | 29 | 0.25 | 0.89 | 0.652 | 0.926 | 1.000 | 993 | tags=14%, list=8%, signal=15% |
| 179 | GOMF\_OXIDOREDUCTASE\_ACTIVITY\_ACTING\_ON\_NAD\_P\_H\_OXYGEN\_AS\_ACCEPTOR |  | 15 | 0.29 | 0.89 | 0.616 | 0.931 | 1.000 | 2195 | tags=33%, list=17%, signal=40% |
| 180 | GOMF\_UBIQUITIN\_LIKE\_PROTEIN\_LIGASE\_ACTIVITY |  | 199 | 0.17 | 0.89 | 0.759 | 0.927 | 1.000 | 3283 | tags=30%, list=25%, signal=40% |
| 181 | GOMF\_LYSOPHOSPHOLIPASE\_ACTIVITY |  | 15 | 0.30 | 0.89 | 0.612 | 0.930 | 1.000 | 2656 | tags=33%, list=21%, signal=42% |
| 182 | GOMF\_NUCLEOSOME\_BINDING |  | 55 | 0.21 | 0.88 | 0.710 | 0.930 | 1.000 | 1362 | tags=15%, list=11%, signal=16% |
| 183 | GOMF\_GLYCOSAMINOGLYCAN\_BINDING |  | 193 | 0.17 | 0.88 | 0.789 | 0.927 | 1.000 | 2550 | tags=22%, list=20%, signal=27% |
| 184 | GOMF\_ORGANIC\_ACID\_BINDING |  | 119 | 0.19 | 0.88 | 0.754 | 0.933 | 1.000 | 3301 | tags=32%, list=26%, signal=43% |
| 185 | GOMF\_CALCIUM\_ACTIVATED\_CATION\_CHANNEL\_ACTIVITY |  | 16 | 0.28 | 0.88 | 0.625 | 0.933 | 1.000 | 1573 | tags=25%, list=12%, signal=28% |
| 186 | GOMF\_DELAYED\_RECTIFIER\_POTASSIUM\_CHANNEL\_ACTIVITY |  | 24 | 0.26 | 0.87 | 0.627 | 0.933 | 1.000 | 496 | tags=13%, list=4%, signal=13% |
| 187 | GOMF\_SIGNALING\_RECEPTOR\_INHIBITOR\_ACTIVITY |  | 23 | 0.26 | 0.87 | 0.642 | 0.932 | 1.000 | 809 | tags=13%, list=6%, signal=14% |
| 188 | GOMF\_PHOSPHOTRANSFERASE\_ACTIVITY\_FOR\_OTHER\_SUBSTITUTED\_PHOSPHATE\_GROUPS |  | 17 | 0.29 | 0.87 | 0.638 | 0.934 | 1.000 | 2648 | tags=35%, list=21%, signal=44% |
| 189 | GOMF\_SH2\_DOMAIN\_BINDING |  | 37 | 0.23 | 0.87 | 0.685 | 0.938 | 1.000 | 3748 | tags=35%, list=29%, signal=49% |
| 190 | GOMF\_SCAVENGER\_RECEPTOR\_ACTIVITY |  | 34 | 0.24 | 0.87 | 0.645 | 0.934 | 1.000 | 960 | tags=12%, list=7%, signal=13% |
| 191 | GOMF\_EXONUCLEASE\_ACTIVITY\_ACTIVE\_WITH\_EITHER\_RIBO\_OR\_DEOXYRIBONUCLEIC\_ACIDS\_AND\_PRODUCING\_5\_PHOSPHOMONOESTERS |  | 44 | 0.22 | 0.86 | 0.725 | 0.945 | 1.000 | 2702 | tags=30%, list=21%, signal=37% |
| 192 | GOMF\_CARBOXYLIC\_ACID\_BINDING |  | 141 | 0.17 | 0.86 | 0.825 | 0.944 | 1.000 | 3701 | tags=34%, list=29%, signal=47% |
| 193 | GOMF\_MONOCARBOXYLIC\_ACID\_BINDING |  | 68 | 0.20 | 0.86 | 0.764 | 0.939 | 1.000 | 3661 | tags=37%, list=28%, signal=51% |
| 194 | GOMF\_GABA\_RECEPTOR\_ACTIVITY |  | 19 | 0.26 | 0.85 | 0.714 | 0.946 | 1.000 | 3350 | tags=37%, list=26%, signal=50% |
| 195 | GOMF\_DNA\_POLYMERASE\_ACTIVITY |  | 24 | 0.25 | 0.85 | 0.660 | 0.949 | 1.000 | 820 | tags=13%, list=6%, signal=13% |
| 196 | GOMF\_GUANYL\_NUCLEOTIDE\_BINDING |  | 303 | 0.16 | 0.84 | 0.952 | 0.963 | 1.000 | 1647 | tags=15%, list=13%, signal=17% |
| 197 | GOMF\_CATALYTIC\_ACTIVITY\_ACTING\_ON\_A\_RRNA |  | 18 | 0.26 | 0.84 | 0.697 | 0.960 | 1.000 | 2107 | tags=22%, list=16%, signal=27% |
| 198 | GOMF\_STEROID\_BINDING |  | 87 | 0.18 | 0.83 | 0.838 | 0.979 | 1.000 | 1728 | tags=14%, list=13%, signal=16% |
| 199 | GOMF\_ADENYLYLTRANSFERASE\_ACTIVITY |  | 23 | 0.25 | 0.83 | 0.716 | 0.976 | 1.000 | 3646 | tags=39%, list=28%, signal=54% |
| 200 | GOMF\_UBIQUITIN\_BINDING |  | 84 | 0.19 | 0.83 | 0.833 | 0.974 | 1.000 | 2354 | tags=23%, list=18%, signal=27% |
| 201 | GOMF\_ION\_GATED\_CHANNEL\_ACTIVITY |  | 25 | 0.24 | 0.82 | 0.739 | 0.972 | 1.000 | 1967 | tags=24%, list=15%, signal=28% |
| 202 | GOMF\_CARGO\_RECEPTOR\_ACTIVITY |  | 64 | 0.19 | 0.82 | 0.790 | 0.973 | 1.000 | 2219 | tags=20%, list=17%, signal=24% |
| 203 | GOMF\_FERROUS\_IRON\_BINDING |  | 20 | 0.26 | 0.82 | 0.740 | 0.973 | 1.000 | 2635 | tags=30%, list=20%, signal=38% |
| 204 | GOMF\_ESTRADIOL\_17\_BETA\_DEHYDROGENASE\_ACTIVITY |  | 16 | 0.26 | 0.81 | 0.742 | 0.987 | 1.000 | 1996 | tags=25%, list=15%, signal=30% |
| 205 | GOMF\_STEROID\_DEHYDROGENASE\_ACTIVITY |  | 29 | 0.22 | 0.80 | 0.779 | 0.996 | 1.000 | 1996 | tags=21%, list=15%, signal=24% |
| 206 | GOMF\_ALCOHOL\_BINDING |  | 76 | 0.18 | 0.80 | 0.843 | 0.997 | 1.000 | 2372 | tags=18%, list=18%, signal=22% |
| 207 | GOMF\_PHOSPHATIDYLCHOLINE\_BINDING |  | 26 | 0.23 | 0.80 | 0.749 | 0.994 | 1.000 | 2734 | tags=35%, list=21%, signal=44% |
| 208 | GOMF\_ATPASE\_ACTIVATOR\_ACTIVITY |  | 24 | 0.23 | 0.78 | 0.801 | 1.000 | 1.000 | 1177 | tags=17%, list=9%, signal=18% |
| 209 | GOMF\_QUATERNARY\_AMMONIUM\_GROUP\_BINDING |  | 32 | 0.21 | 0.78 | 0.818 | 1.000 | 1.000 | 2734 | tags=34%, list=21%, signal=44% |
| 210 | GOMF\_TRANSFERASE\_ACTIVITY\_TRANSFERRING\_SULPHUR\_CONTAINING\_GROUPS |  | 51 | 0.19 | 0.77 | 0.856 | 1.000 | 1.000 | 3497 | tags=35%, list=27%, signal=48% |
| 211 | GOMF\_MYOSIN\_BINDING |  | 61 | 0.19 | 0.77 | 0.896 | 1.000 | 1.000 | 3119 | tags=30%, list=24%, signal=39% |
| 212 | GOMF\_NUCLEOBASE\_CONTAINING\_COMPOUND\_TRANSMEMBRANE\_TRANSPORTER\_ACTIVITY |  | 35 | 0.21 | 0.77 | 0.850 | 1.000 | 1.000 | 395 | tags=9%, list=3%, signal=9% |
| 213 | GOMF\_CYTOKINE\_BINDING |  | 123 | 0.16 | 0.77 | 0.957 | 1.000 | 1.000 | 2222 | tags=20%, list=17%, signal=23% |
| 214 | GOMF\_NUCLEOSOMAL\_DNA\_BINDING |  | 34 | 0.21 | 0.77 | 0.822 | 1.000 | 1.000 | 1362 | tags=15%, list=11%, signal=16% |
| 215 | GOMF\_CYSTEINE\_TYPE\_ENDOPEPTIDASE\_INHIBITOR\_ACTIVITY |  | 44 | 0.19 | 0.76 | 0.899 | 1.000 | 1.000 | 2519 | tags=23%, list=20%, signal=28% |
| 216 | GOMF\_NADPLUS\_NUCLEOSIDASE\_ACTIVITY |  | 23 | 0.22 | 0.75 | 0.833 | 1.000 | 1.000 | 802 | tags=13%, list=6%, signal=14% |
| 217 | GOMF\_PHOSPHATIDYLSERINE\_BINDING |  | 45 | 0.19 | 0.75 | 0.893 | 1.000 | 1.000 | 730 | tags=11%, list=6%, signal=12% |
| 218 | GOMF\_ASPARTIC\_TYPE\_PEPTIDASE\_ACTIVITY |  | 16 | 0.25 | 0.74 | 0.812 | 1.000 | 1.000 | 666 | tags=13%, list=5%, signal=13% |
| 219 | GOMF\_ENDOPEPTIDASE\_REGULATOR\_ACTIVITY |  | 141 | 0.15 | 0.74 | 0.983 | 1.000 | 1.000 | 3389 | tags=28%, list=26%, signal=37% |
| 220 | GOMF\_DYNEIN\_COMPLEX\_BINDING |  | 20 | 0.23 | 0.74 | 0.839 | 1.000 | 1.000 | 1874 | tags=20%, list=15%, signal=23% |
| 221 | GOMF\_APOLIPOPROTEIN\_BINDING |  | 17 | 0.24 | 0.74 | 0.829 | 1.000 | 1.000 | 1491 | tags=18%, list=12%, signal=20% |
| 222 | GOMF\_CYSTEINE\_TYPE\_ENDOPEPTIDASE\_INHIBITOR\_ACTIVITY\_INVOLVED\_IN\_APOPTOTIC\_PROCESS |  | 19 | 0.22 | 0.73 | 0.864 | 1.000 | 1.000 | 2161 | tags=26%, list=17%, signal=32% |
| 223 | GOMF\_STEROID\_DEHYDROGENASE\_ACTIVITY\_ACTING\_ON\_THE\_CH\_OH\_GROUP\_OF\_DONORS\_NAD\_OR\_NADP\_AS\_ACCEPTOR |  | 25 | 0.21 | 0.73 | 0.870 | 1.000 | 1.000 | 1996 | tags=20%, list=15%, signal=24% |
| 224 | GOMF\_TUMOR\_NECROSIS\_FACTOR\_RECEPTOR\_BINDING |  | 29 | 0.20 | 0.72 | 0.891 | 1.000 | 1.000 | 3107 | tags=28%, list=24%, signal=36% |
| 225 | GOMF\_TELOMERIC\_DNA\_BINDING |  | 36 | 0.19 | 0.71 | 0.933 | 1.000 | 1.000 | 1340 | tags=14%, list=10%, signal=15% |
| 226 | GOMF\_VOLTAGE\_GATED\_ANION\_CHANNEL\_ACTIVITY |  | 15 | 0.24 | 0.71 | 0.862 | 1.000 | 1.000 | 2734 | tags=33%, list=21%, signal=42% |
| 227 | GOMF\_EXONUCLEASE\_ACTIVITY |  | 61 | 0.17 | 0.71 | 0.963 | 1.000 | 1.000 | 2702 | tags=26%, list=21%, signal=33% |
| 228 | GOMF\_SYNTAXIN\_1\_BINDING |  | 16 | 0.23 | 0.70 | 0.868 | 1.000 | 1.000 | 908 | tags=13%, list=7%, signal=13% |
| 229 | GOMF\_SUGAR\_TRANSMEMBRANE\_TRANSPORTER\_ACTIVITY |  | 17 | 0.22 | 0.70 | 0.909 | 1.000 | 1.000 | 1634 | tags=18%, list=13%, signal=20% |
| 230 | GOMF\_IMMUNE\_RECEPTOR\_ACTIVITY |  | 128 | 0.14 | 0.69 | 0.994 | 1.000 | 1.000 | 3421 | tags=28%, list=27%, signal=38% |
| 231 | GOMF\_IONOTROPIC\_GLUTAMATE\_RECEPTOR\_BINDING |  | 15 | 0.23 | 0.68 | 0.879 | 1.000 | 1.000 | 908 | tags=13%, list=7%, signal=14% |
| 232 | GOMF\_DYNEIN\_LIGHT\_INTERMEDIATE\_CHAIN\_BINDING |  | 16 | 0.23 | 0.68 | 0.902 | 1.000 | 1.000 | 1578 | tags=19%, list=12%, signal=21% |
| 233 | GOMF\_RETINOIC\_ACID\_BINDING |  | 17 | 0.22 | 0.67 | 0.914 | 1.000 | 1.000 | 3606 | tags=47%, list=28%, signal=65% |
| 234 | GOMF\_ACTIN\_MONOMER\_BINDING |  | 24 | 0.20 | 0.67 | 0.938 | 1.000 | 1.000 | 3867 | tags=38%, list=30%, signal=53% |
| 235 | GOMF\_C\_C\_CHEMOKINE\_BINDING |  | 24 | 0.19 | 0.66 | 0.950 | 1.000 | 1.000 | 3421 | tags=38%, list=27%, signal=51% |
| 236 | GOMF\_TRNA\_METHYLTRANSFERASE\_ACTIVITY |  | 21 | 0.19 | 0.64 | 0.927 | 1.000 | 1.000 | 946 | tags=10%, list=7%, signal=10% |
| 237 | GOMF\_O\_ACYLTRANSFERASE\_ACTIVITY |  | 35 | 0.17 | 0.63 | 0.980 | 1.000 | 1.000 | 1811 | tags=17%, list=14%, signal=20% |
| 238 | GOMF\_VOLTAGE\_GATED\_CALCIUM\_CHANNEL\_ACTIVITY |  | 32 | 0.17 | 0.61 | 0.978 | 1.000 | 1.000 | 4031 | tags=34%, list=31%, signal=50% |
| 239 | GOMF\_COMPLEMENT\_BINDING |  | 18 | 0.20 | 0.61 | 0.948 | 1.000 | 1.000 | 3918 | tags=44%, list=30%, signal=64% |
| 240 | GOMF\_STEROL\_BINDING |  | 52 | 0.15 | 0.61 | 0.990 | 1.000 | 1.000 | 2317 | tags=15%, list=18%, signal=19% |
| 241 | GOMF\_INTRACELLULAR\_LIGAND\_GATED\_ION\_CHANNEL\_ACTIVITY |  | 24 | 0.18 | 0.60 | 0.966 | 1.000 | 1.000 | 3517 | tags=29%, list=27%, signal=40% |
| 242 | GOMF\_FIBROBLAST\_GROWTH\_FACTOR\_RECEPTOR\_BINDING |  | 22 | 0.18 | 0.60 | 0.963 | 1.000 | 1.000 | 1833 | tags=18%, list=14%, signal=21% |
| 243 | GOMF\_LIGASE\_ACTIVITY\_FORMING\_CARBON\_SULFUR\_BONDS |  | 32 | 0.16 | 0.59 | 0.981 | 1.000 | 1.000 | 3525 | tags=31%, list=27%, signal=43% |
| 244 | GOMF\_ORGANIC\_HYDROXY\_COMPOUND\_TRANSMEMBRANE\_TRANSPORTER\_ACTIVITY |  | 38 | 0.15 | 0.58 | 0.995 | 1.000 | 1.000 | 2051 | tags=16%, list=16%, signal=19% |
| 245 | GOMF\_MHC\_PROTEIN\_BINDING |  | 33 | 0.16 | 0.58 | 0.988 | 1.000 | 1.000 | 4527 | tags=48%, list=35%, signal=75% |
| 246 | GOMF\_SULFOTRANSFERASE\_ACTIVITY |  | 36 | 0.15 | 0.55 | 0.980 | 1.000 | 1.000 | 3497 | tags=33%, list=27%, signal=46% |
| 247 | GOMF\_CHOLESTEROL\_BINDING |  | 43 | 0.14 | 0.54 | 1.000 | 1.000 | 1.000 | 2317 | tags=14%, list=18%, signal=17% |
| 248 | GOMF\_ACETYLCHOLINE\_GATED\_CATION\_SELECTIVE\_CHANNEL\_ACTIVITY |  | 16 | 0.17 | 0.53 | 0.991 | 1.000 | 1.000 | 1393 | tags=13%, list=11%, signal=14% |
| 249 | GOMF\_MICROFILAMENT\_MOTOR\_ACTIVITY |  | 27 | 0.14 | 0.50 | 1.000 | 1.000 | 1.000 | 11042 | tags=100%, list=86%, signal=693% |
| 250 | GOMF\_CARBOHYDRATE\_TRANSMEMBRANE\_TRANSPORTER\_ACTIVITY |  | 26 | 0.14 | 0.48 | 1.000 | 1.000 | 1.000 | 1634 | tags=12%, list=13%, signal=13% |
| 251 | GOMF\_STEROL\_TRANSFER\_ACTIVITY |  | 20 | 0.14 | 0.45 | 1.000 | 0.999 | 1.000 | 2942 | tags=25%, list=23%, signal=32% |
Table: Gene sets enriched in phenotype **OA (10 samples) **[plain text format]****
